# Morphological examination and phylogenetic analyses reveal two new species in *Pleurotheciaceae* from China

**DOI:** 10.3897/mycokeys.136.199476

**Published:** 2026-07-15

**Authors:** Yu Yang, Xing-Juan Xiao, Yan-Ling Yang, Yong-Zhong Lu, Kevin D. Hyde, Yuan-Pin Xiao, Ying Liu, Zhen-Ling Chen, Gui-Li Zhao

**Affiliations:** 1 School of Chemical Engineering, Guizhou Institute of Technology, Guiyang 550025, China Innovative Institute for Plant Health / Key Laboratory of Green Prevention and Control on Fruits and Vegetables in South China, Ministry of Agriculture and Rural Affairs, Zhongkai University of Agriculture and Engineering Guangzhou China; 2 School of Food and Pharmaceutical Engineering, Guizhou Institute of Technology, Guiyang 550025, China Guizhou Key Laboratory of Agricultural Microbiology, Guizhou Academy of Agricultural Sciences Guiyang China; 3 Guizhou Key Laboratory of Agricultural Microbiology, Guizhou Academy of Agricultural Sciences, Guiyang 550009, China Center of Excellence in Fungal Research, Mae Fah Luang University Chiang Rai Thailand; 4 Innovative Institute for Plant Health / Key Laboratory of Green Prevention and Control on Fruits and Vegetables in South China, Ministry of Agriculture and Rural Affairs, Zhongkai University of Agriculture and Engineering, Guangzhou, 510225, China School of Chemical Engineering, Guizhou Institute of Technology Guiyang China; 5 Center of Excellence in Fungal Research, Mae Fah Luang University, Chiang Rai, 57100, Thailand School of Food and Pharmaceutical Engineering, Guizhou Institute of Technology Guiyang China

**Keywords:** 2 new taxa, asexual morph, hyphomycetes, *
Sordariomycetes
*, taxonomy

## Abstract

During an ongoing investigation of saprobic fungi in southern China, two hyphomycetous fungi of *Pleurotheciaceae* were collected and isolated from decaying wood in freshwater and terrestrial habitats. Morphology and multi-locus phylogenetic analyses of a combined LSU, ITS, SSU and *rpb*2 sequence dataset, showed that *Pleurotheciella guizhouensis* and *Pleurothecium guangxiense* are introduced as new species, which are introduced here. Both species have macronematous mononematous conidiophores, and polyblastic conidiogenous cells with hyaline conidia, and a sympodial arrangement on cylindrical denticles. Morphological comparisons and phylogenetic analyses support the recognition of both taxa as distinct species within *Pleurotheciaceae*, further expanding the known diversity of *Pleurotheciaceae* in China.

## Introduction

*Pleurotheciaceae* was introduced by Réblová et al. (2016), with *Pleurothecium* as the type genus, and belongs to *Pleurotheciales* within *Sordariomycetes* (Hyde et al. 2024). The family currently comprises 16 genera and more than 100 accepted species occurring in both aquatic and terrestrial habitats (Réblová et al. 2016; He et al. 2024; Wang et al. 2024; Xu et al. 2025a; Zhang et al. 2025c). Both sexual and asexual morphs have been reported, although most taxa are primarily known from aquatic asexual morphs associated with decaying plant substrates, highlighting the ecological and potential resource value of these fungi (Réblová et al. 2016; Luo et al. 2018; Liu et al. 2026; Wang et al. 2025a; Zhang et al. 2025c). A comprehensive overview of freshwater species of *Pleurotheciaceae*, including host and geographic distribution data, was provided by Wang et al. (2024). Among the genera currently accepted in *Pleurotheciaceae*, *Pleurothecium* and *Pleurotheciella* are commonly reported from both aquatic and terrestrial habitats and share similar morphological characteristics (Réblová et al. 2016; Dong et al. 2021; Xu et al. 2025a; Zhang et al. 2025c).

*Pleurothecium* was established by Franz Xaver Rudolf von Höhnel (1919), with *P. recurvatum* designated as the type species. The sexual morph of *Pleurothecium* resembles that of *Chaetosphaeria*, but the two genera differ in their asexual morph (He et al. 2024; Lin et al. 2025). The asexual morphs of *Pleurothecium* are characterized by distinct brown macronematous conidiophores, polyblastic conidiogenous cells that extend sympodially and bear denticles, and solitary, unicellular or septate, hyaline to pigmented conidia (Fernández et al. 1999; Réblová et al. 2012; Luo et al. 2019; He et al. 2024). Currently, 20 species are accepted in this genus (Lin et al. 2025; Wang et al. 2025a; Xu et al. 2025a), with most taxa reported from aquatic habitats in China (Jayawardena et al. 2022; He et al. 2024; Wang et al. 2025a; Xu et al. 2025a). All known species of *Pleurothecium* possess hyphomycetous asexual morphs, whereas sexual morphs have only been reported for *P. recurvatum* and *P. semifecundum* (Réblová et al. 2012).

Réblová et al. (2012) introduced the genus *Pleurotheciella* to accommodate two new pleurothecium-like species from submerged decaying wood, *Pla. centenaria* and *Pla. rivularia*, with the latter designated as the type species. The genus is characterized by a chaetosphaeria-like sexual morph with perithecial, astromatic ascomata, unitunicate asci, and hyaline septate ascospores (Réblová et al. 2012). Its asexual morph is dactylaria-like, producing polyblastic conidia sympodially on a rachis or denticles (Réblová et al. 2012; Abdel-Aziz et al. 2020; He et al. 2024; Visagie et al. 2024; Wang et al. 2024; Li et al. 2025; Wang et al. 2025a). Currently, 31 species are accepted in *Pleurotheciella*, which has a worldwide distribution, with most taxa reported from freshwater habitats (Wang et al. 2024; Li et al. 2025; Xu et al. 2025b; Wang et al. 2025a).

In this study, two hyphomycetous taxa belonging to *Pleurotheciaceae* were collected and isolated from freshwater and terrestrial habitats in southern China and are introduced as two novel species, *Pleurotheciella guizhouensis* and *Pleurothecium guangxiense*. The establishment of these taxa is based on a taxonomic approach integrating morphological characteristics and phylogenetic analyses of a combined LSU, ITS, SSU, and *rpb*2 sequence dataset. The introduction of the new species follows the guidelines of Maharachchikumbura et al. (2021) and Aime et al. (2021).

## Materials and methods

### Specimen collection, examination, and isolation

Decaying wood samples were collected from forests in Guangxi and Guizhou provinces, China. The samples were packed in sealable plastic bags, with associated metadata, including collection date, locality, and host (Rathnayaka et al. 2025), and taken to the laboratory. Fungal colonies on the host surface were examined using stereomicroscopes (Nikon SMZ 745 and SMZ 800N, Tokyo, Japan). Micromorphological features, including mycelia, conidiophores, conidiogenous cells, and conidia, were observed and photographed using a Nikon DS-Ri2 digital camera fitted to a Nikon ECLIPSE Ni compound microscope (Nikon, Japan). Morphological measurements were made using Tarosoft Image Frame Work v.0.9.7. Photoplates were processed and combined using Adobe Photoshop CC 2019 (Adobe Systems, USA).

Single-spore isolations were made on potato dextrose agar (PDA; 39 g/L distilled water, Difco potato dextrose agar), and germinated spores were individually transferred to PDA following the method described by Senanayake et al. (2020). Dried specimens were deposited in the Herbarium of Cryptogams, Kunming Institute of Botany, Chinese Academy of Sciences (HKAS), Kunming, China. Pure cultures were deposited in the Guizhou Culture Collection, China (**GZCC**). Index Fungorum numbers were registered for the new taxa according to the instructions in Index Fungorum (2026) and new species are established following recommendations outline by Jeewon and Hyde (2016).

### DNA extraction, PCR amplification, and sequencing

Germinated spores were cultured on PDA at room temperature for two months. Fungal mycelia were scraped from the culture surface with a sterile scalpel and transferred to 1.5 mL microcentrifuge tubes for genomic DNA extraction. Genomic DNA was extracted using a Trelief™ Plant Genomic DNA Kit (Beijing TsingKe Biotech Co., Beijing, China) following the manufacturer’s instructions. Four partial gene regions, including the small subunit ribosomal DNA (SSU), internal transcribed spacer (ITS), large subunit ribosomal DNA (LSU), and RNA polymerase II second largest subunit (*rpb*2), were amplified by polymerase chain reaction (PCR). The primer pairs used were NS1/NS4 (White et al. 1990) for SSU, ITS5/ITS4 (White et al. 1990) for ITS, LR0R/LR5 (Vilgalys and Hester 1990) for LSU, and fRPB2-5F/fRPB2-7cR (Liu et al. 1999) for *rpb*2, respectively. PCR amplification conditions consisted of an initial denaturation at 98 °C for 2 min, followed by 35 cycles of denaturation at 98 °C for 10 s, annealing at 55 °C for 1 min, and extension at 72 °C for 30 s, with a final extension at 72 °C for 2 min. Purification and sequencing of PCR products were carried out by Tsingke Biotechnology Co., Ltd. (Beijing, China).

### Phylogenetic analyses

Newly generated sequences were checked in BioEdit v.7.0.5.3 (Hall 1999) and assembled using SeqMan v.7.0.0 (DNASTAR, Madison, WI, USA). Closely related taxa for phylogenetic analyses were selected based on BLASTn searches in NCBI GenBank (https://blast.ncbi.nlm.nih.gov/Blast.cgi) and relevant publications. Sequence alignments for each locus were generated using the online version of MAFFT v.7.307 (https://mafft.cbrc.jp/alignment/server/; Katoh et al. 2019). Ambiguously aligned regions and uninformative positions were trimmed using TrimAl v.1.2 with the “gt 0.6” option (Capella-Gutiérrez et al. 2009). Multi-gene datasets were concatenated using SequenceMatrix v.1.7.8 (Vaidya et al. 2011) and analyzed using maximum likelihood (ML) and Bayesian inference (BI). Newly generated sequences were deposited in GenBank (Table 1). ML and BI analyses were performed through the CIPRES Science Gateway V.3.3 (https://www.phylo.org/portal2/home.action; Miller et al. 2010). ML analysis was conducted using RAxML-HPC v.8.2.12 under the GTRGAMMA substitution model with rapid bootstrap analysis and 1000 bootstrap replicates (Stamatakis 2014). BI analysis was performed using MrBayes v.3.2.7a on XSEDE. The best-fit substitution model was determined using MrModeltest v.2.3 (Nylander 2004). Four Markov chains were run simultaneously for 50,000,000 generations, with trees sampled every 100 generations until the average standard deviation of split frequencies fell below 0.01. The first 25% of sampled trees were discarded as burn-in, and the remaining trees were used to calculate posterior probabilities (Larget and Simon 1999). Phylogenetic trees were visualized using FigTree v.1.4.4 (Rambaut 2014), and the final layouts were edited in Adobe Illustrator CS6 (Adobe Systems, San Jose, CA, USA).

**Table 1.** The table below lists the taxa used in this study, with their respective GenBank accession numbers.

**Taxon**	**Strain**	**GenBank accessions**			
		**LSU**	**ITS**	**SSU**	***rpb*2**
*Adelosphaeria catenata*	CBS 138679^T^	KT278707	KT278721	KT278692	KT278743
*Anapleurothecium botulisporum*	CBS 132713^T^	KY853483	KY853423	-	-
*Ascolacicola mitriformis*	HKUCC 3706	AF132324	-	-	-
*Ascolacicola sawadae*	SS 00051	HQ446363	-	HQ446283	HQ446418
*Coleodictyospora muriformis*	MFLUCC 18-1243^T^	NG_148991	NR_182540	NG_148852	-
*Dematipyriforma aquilaria*	CGMCC 3.17268^T^	KJ138623	KJ138621	KJ138622	-
*Helicoascotaiwania farinose*	DAOM 241947	JQ429230	JQ429145	-	-
*Melanotrigonum ovale*	CBS 138743^T^	KT278709	KT278724	KT278696	KT278745
*Monotosporella setosa*	HKUCC 3713	AF132334	-	-	-
*Neomonodictys muriformis*	MFLUCC 16-1136^T^	NG_068916	NR_168231	-	-
*Phaeoisaria dalbergiae*	CBS 148440^T^	NG_081319	NR_175205	NG_148880	OK651159
*Phaeoisaria fasciculata*	CBS 127885^T^	KT278705	KT278719	KT278693	KT278741
*Phragmocephala stemphylioides*	DAOM 673211	KT278717	KT278730	-	-
*Pleurotheciella acericola*	CBS 150809^T^	PP872412	PP872401	-	PP874919
*Pleurotheciella aquatica*	MFLUCC 17-0464^T^	MF399253	MF399236	MF399220	MF401405
*Pleurotheciella aquatica*	KUNCC 24-19069	PV264840	PV264831	PV335232	PV298273
*Pleurotheciella atroseptata*	HKAS 146437	PV264842	PV264833	PV335234	-
*Pleurotheciella atroseptata*	HKAS 146441	PV264843	PV264834	PV335235	-
*Pleurotheciella bambusisiliquosa*	KUNCC 24-18079	PQ152643	PQ168255	-	-
*Pleurotheciella bambusisiliquosa*	KUNCC 24-18078^T^	PQ152642	PQ168254	PQ218271	-
*Pleurotheciella brachyspora*	CGMCC 3.25435^T^	OR600969	OR589321	PP049532	PP068773
*Pleurotheciella brachyspora*	KUNCC 24-18257	PQ650088	PQ644520	-	-
*Pleurotheciella brevis*	HKAS 105178^T^	-	PQ898774	-	-
*Pleurotheciella centenaria*	DAOM 229631^T^	JQ429234	JQ429151	JQ429246	JQ429265
*Pleurotheciella centenaria*	MFLUCC 17-0114	MK835849	-	-	MN194027
*Pleurotheciella curvata*	CGMCC 3.27020	-	PP049502	PQ844513	PQ824483
*Pleurotheciella dimorphospora*	KUMCC 20-0185^T^	MW981444	MW981446	MW981454	MZ509665
*Pleurotheciella erumpens*	CBS 142447^T^	MN699435	MN699406	MN699387	MN704311
*Pleurotheciella erumpens*	KUNCC 24-19088	PV264844	PV264835	PV335236	PV298275
*Pleurotheciella fusiformis*	MFLUCC 17-0113^T^	MF399250	MF399233	MF399218	MF401403
*Pleurotheciella fusiformis*	MFLUCC 17-0115	MF399249	MF399232	MF399217	MF401402
*Pleurotheciella ganzhouensis*	JAUCC6079^T^	OR853422	OR853417	OR853426	PP078759
*Pleurotheciella ganzhouensis*	JAUCC6678	PP800214	PP800192	PP801261	PP816289
*Pleurotheciella guangxiensis*	KUNCC 24-18269	PQ650092	PQ644524	PQ844510	-
***Pleurotheciella guizhouensis***	**GZCC 23-0747** ^T^	**PZ394742**	**PZ394735**	**PZ394749**	**PZ437644**
*Pleurotheciella guttulata*	KUMCC 15-0296^T^	MF399257	MF399240	MF399223	MF401409
*Pleurotheciella guttulata*	KUMCC 15-044	MF399256	MF399239	MF399222	MF401408
*Pleurotheciella hyalospora*	GZCC 22-2018^T^	OQ002371	OQ002374	OQ002377	OP999221
*Pleurotheciella hyalospora*	GZCC 22-2023	OQ002370	OQ002373	OQ002376	OP999220
*Pleurotheciella irregularis*	JAUCC6080^T^	OR853423	OR853418	PP801258	PP816286
*Pleurotheciella irregularis*	JAUCC6679	-	PP800193	PP801262	-
*Pleurotheciella krabiensis*	MFLUCC 16-0852^T^	MG837013	MG837018	MG837023	-
*Pleurotheciella krabiensis*	MFLUCC 18-0858	MG837014	MG837019	MG837024	-
*Pleurotheciella longidenticulata*	CGMCC 3.27018^T^	PP049513	PP049496	PP049531	PP068776
*Pleurotheciella lunata*	MFLUCC 17-0111^T^	MF399255	MF399238	MF399221	MF401407
*Pleurotheciella lunata*	S-426	MK835847	MK878378	MK834782	-
*Pleurotheciella nilotica*	MD 1317^T^	KX611344	-	MN356449	-
*Pleurotheciella nilotica*	KUMCC 19-0214	MT559087	MT555416	MT559095	-
*Pleurotheciella obliqua*	CGMCC 3.27019	PP049514	PP049497	PP049533	PP068774
*Pleurotheciella obliqua*	KUNCC 23-16569	PP049515	PP049498	PP049534	PP068777
*Pleurotheciella rivularia*	CBS 125238^T^	JQ429232	JQ429160	JQ429244	JQ429263
*Pleurotheciella rivularia*	CBS 125237	JQ429233	JQ429161	JQ429245	JQ429264
*Pleurotheciella saprophytica*	MFLUCC 16-1251^T^	MF399258	MF399241	MF399224	MF401410
*Pleurotheciella submersa*	MFLUCC 17-1709^T^	MF399260	MF399243	MF399226	MF401412
*Pleurotheciella submersa*	MFLUCC 17-0456	MF399261	MF399244	MF399227	MF401413
*Pleurotheciella sympodia*	KUMCC 19-0213^T^	MT555426	MT555420	-	-
*Pleurotheciella sympodia*	MFLUCC 18-1408	MW981652	MW981644	-	-
*Pleurotheciella tropica*	MFLUCC 16-0867^T^	MG837015	MG837020	MG837025	-
*Pleurotheciella uniseptata*	S-936	MK835846	MK878377	MK834781	MN194025
*Pleurotheciella uniseptata*	KUNCC 23-16863	PQ650089	PQ644521	PQ844507	PQ824469
*Pleurotheciella uniseptata*	DAOM 673210^T^	KT278716	KT278729	-	-
*Pleurotheciella xizangensis*	KUNCC 25-19266	PV264845	PV264836	PV335237	PV582134
*Pleurotheciella yunnanensis*	KUNCC 23-13328^T^	PP095383	OR234682	PP095382	PP131261
*Pleurotheciella yunnanensis*	KUNCC 23-13682	PP095381	PP095384	PP095385	PP131262
*Pleurothecium aquaticum*	MFLUCC 17-1331^T^	MF399263	MF399245	-	-
*Pleurothecium aquaticum*	MFLUCC 21-0148	OM654772	OM654775	OM654807	-
*Pleurothecium aquisubtropicum*	GZCC 21-0670^T^	OM339433	OM339436	-	-
*Pleurothecium aseptatum*	GZCC 22-2019^T^	OQ002372	OQ002375	-	-
*Pleurothecium brunius*	SCF-2023a^T^	OQ799377	OQ799378	OQ799376	-
*Pleurothecium floriforme*	MFLUCC 15-0628^T^	KY697277	KY697281	NG_063634	-
***Pleurothecium guangxiense***	**GZCC 26**-**0126**^T^	**PZ394740**	**PZ394733**	**PZ394747**	**PZ437642**
***Pleurothecium guangxiense***	**GZCC 26**-**0127**	**PZ394741**	**PZ394734**	**PZ394748**	**PZ437643**
*Pleurothecium guttulatum*	IFRD 9203^T^	MT559115	MT555415	MT559089	-
*Pleurothecium hainanense*	GZCC 22-2021^T^	OP748931	OP748934	-	-
*Pleurothecium hyalosporum*	HKAS 105128^T^	PQ898780	PQ898744	PQ898814	-
*Pleurothecium jiangxiense*	JAUCC6077^T^	OR853420	OR853415	OR853425	PP078757
*Pleurothecium jiangxiense*	JAUCC6676	PP800212	PP800190	PP801260	PP816288
*Pleurothecium lignicola*	JAUCC 7034^T^	PQ443974	PQ443962	PQ444007	PQ569768
*Pleurothecium lignicola*	JAUCC 7035	PQ443975	PQ443963	PQ444008	-
*Pleurothecium pisiformis*	KUNCC 24-19085	PV264846	PV264837	PV335238	-
*Pleurothecium pulneyense*	MFLUCC 16-1293	MF399262	-	MF399228	MF401414
*Pleurothecium recurvatum*	CBS 131272	JQ429237	JQ429149	JQ429251	JQ429268
*Pleurothecium recurvatum*	CBS 131646	JQ429236	JQ429150	JQ429250	-
*Pleurothecium semifecundum*	CBS 131271^T^	JQ429240	JQ429159	JQ429254	JQ429270
*Pleurothecium semifecundum*	CBS 131482	JQ429239	JQ429158	JQ429253	-
*Pleurothecium takense*	BBH 49602	OQ121949	OQ121931	OQ121940	OQ116754
*Pseudosaprodesmium cocois*	MFLU 23-0225^T^	OR438864	OR438401	NG_244179	-
*Rhexoacrodictys dematiospora*	KUMCC 18-0059^T^	MW981647	NR_182542	NG_148854	-
*Sterigmatobotrys rudis*	KUNCC 24-18066	PQ455306	PQ218140	PV643446	-
*Adelosphaeria catenata*	CBS 138679^T^	KT278707	KT278721	KT278692	KT278743

Note: “T” represents the ex-type strain. “-” indicates data unavailability. Newly generated sequences are represented in bold.

## Taxonomy and results

### Phylogenetic analyses

**Figure 1.**
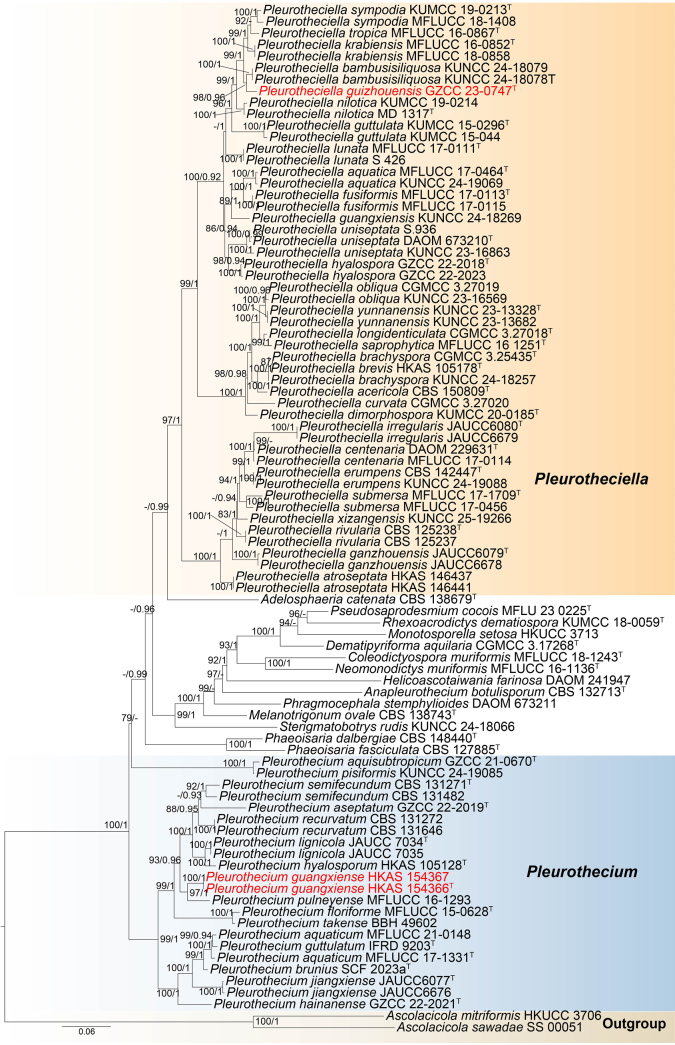
The phylogenetic tree inferred using Maximum Likelihood (ML) in RAxML, incorporating combined sequence data from six nuclear loci, including LSU, ITS, SSU, and *rpb*2. *Ascolacicola mitriformis* (HKUCC 3706) and *A. sawadae* (SS 00051) were designated as outgroup taxa. Node support values are indicated on the phylogram; ML bootstrap values ≥ 75% and Bayesian posterior probabilities (PP)≥0.90 are considered significant. Newly generated sequences are highlighted in red.

Phylogenetic analyses were conducted using LSU, ITS, SSU, and *rpb*2 sequence data to determine the phylogenetic placement of the newly collected taxa. The combined dataset comprised sequences from 89 isolates, with *Ascolacicola mitriformis* (HKUCC 3706) and *A. sawadae* (SS 00051) selected as the outgroup taxa. The concatenated sequence matrix included ITS (1–552 bp), LSU (553–1,383 bp), *rpb*2 (1,384–2,290 bp), and SSU (2,291–3,243 bp). The best-scoring ML tree generated by RAxML (Fig. 1) had a final likelihood value of –27459.387512. The estimated base frequencies were A = 0.236550, C = 0.257775, G = 0.288144, and T = 0.217530; substitution rates were AC = 1.437571, AG = 3.364721, AT = 1.637114, CG = 1.108144, CT = 7.152794, and GT = 1.000000. The gamma distribution shape parameter (α) was estimated as 0.195408. Maximum likelihood and Bayesian inference analyses generated similar tree topologies.

Phylogenetic analyses showed that the 16 genera within *Pleurotheciaceae (Pleurotheciales)* were resolved as distinct lineages (Fig. 1). The three newly collected isolates were resolved into two independent lineages. Strain GZCC 23-0747 of *Pleurotheciella guizhouensis* formed a strongly supported sister clade to *Pla. bambusisiliquosa* (KUNCC 24-18078 and KUNCC 24-18079), with 98% ML bootstrap support and 0.96 Bayesian posterior probability (BYPP; Fig. 1). Similarly, strains GZCC 26-0126 and GZCC 26-0127 of *Pleurothecium guangxiense* formed a distinct sister lineage to *P. pulneyense* (MFLUCC 16-1293), with 97% ML bootstrap support and 1.00 BYPP.

#### Pleurotheciella guizhouensis

Taxon classificationFungiPleurothecialesPleurotheciaceae

Y. Yang, G.L. Zhao & X.J. Xiao sp. nov.

157F5084-467F-555D-B659-CA73C738B7B8

Index Fungorum: IF905503

Fig. 2

##### Etymology.

Refers to the type locality (Guizhou Province), where the holotype was collected.

**Figure 2.**
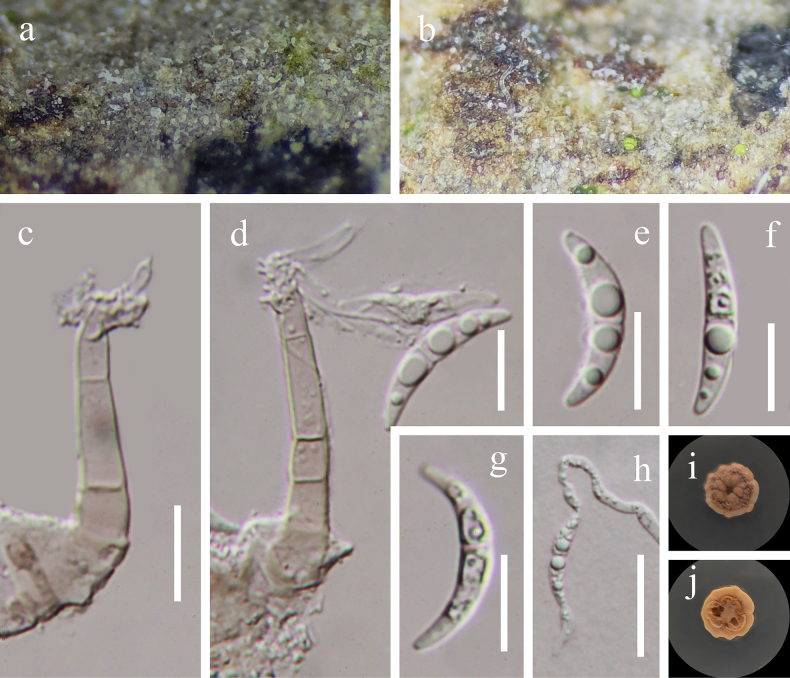
*Pleurotheciella guizhouensis* (HKAS 154362, holotype) **a, b**. Colonies on the host surface; **c, d**. Conidiophores, conidiogenous cells and conidia; **e–g**. Conidia; **h**. Germinated conidium; **i, j**. Surface and reverse colonies on PDA. Scale bars: 20 μm (**h**); 10 μm (**c–g**).

##### Holotype.

HKAS 154362.

##### Description.

***Saprobic*** on dead wood in a freshwater habitat. ***Sexual morph*** Undetermined. ***Asexual morph*** Hyphomycetous. ***Colonies*** on natural substrate superficial, effuse, solitary, white to hyaline. ***Mycelium*** partly immersed, partly superficial, branched, septate, light brown to hyaline hyphae. ***Conidiophores*** 23–53 × 3.5–5.5 μm (*x̄* = 38 × 4.7 μm, n = 25), macronematous, mononematous, single, unbranched, septate, cylindrical, straight or slightly curved, light brown to hyaline, smooth. ***Conidiogenous cells*** 8.5–14.5 × 2–3.6 µm (*x̄* = 11.5 × 2.9 µm, n = 20), polyblastic, polyphialidic, integrated, terminal, cylindrical, hyaline, forming conidia sympodially on cylindrical denticles or wart. ***Conidia*** 19–23 µm × 2.5–3.7 µm (*x̄* = 21 × 3.1 µm, n = 30), narrowly fusiform or meniscus, solitary, hyaline, guttulate, straight or arcuate, 0–1-septate, pointed at one end, the other round and wide in the middle, smooth-walled.

##### Culture characteristics.

Conidium germinated on PDA at 28 °C within 24 h from single-spore isolation. After two months of incubation at room temperature, the colony reached a diameter of 15 mm, with rough, dry, circular, irregular edges, dense in texture, with a brown central region and a pale brown outer margin, and the reverse was pale brown.

##### Material examined.

China • Guizhou Province, Qiandongnan Miao and Dong Autonomous Prefecture, Zhenyuan County (27°14'40"N, 108°18'30"E, altitude 625 m), on submerged decaying wood in a stream, 3 May 2025, Xing-Juan Xiao, GS7 (HKAS 154362, holotype), ex-type culture GZCC 23-0747.

##### Notes.

Phylogenetically, *Pleurotheciella guizhouensis* formed a distinct monophyletic lineage sister to *Pla. bambusisiliquosa*, with 98% ML/0.96 BYPP support (98% ML/0.96 BYPP; Fig. 1). Pairwise nucleotide comparisons showed that *Pla. guizhouensis* (GZCC 23-0747, ex-type) shared 517/525 bp (98.5%) sequence similarity in the ITS regions, 794/800 bp (99.3%) in the LSU region, and 736/738 bp (99.7%) in the SSU region with *Pla. bambusisiliquosa* (KUNCC 24-18078, ex-type), including gaps. In addition, the *rpb*2 sequence of KUNCC 24-18078 was unavailable. Morphologically, *Pla. bambusisiliquosa* is characterized by solitary or clustered conidiophores in groups of 2–4, whereas the new species differs in having exclusively solitary, shorter conidiophores (23–53 × 3.5–5.5 µm vs. 142–177 × 4–7 µm), shorter conidiogenous cells (8.5–14.5 × 2–3.6 µm vs. 26–40 × 3–4 µm), and longer conidia (19–23 × 2.5–3.7 µm vs. 14–19 × 3–5 µm) (Xu et al. 2025b). Therefore, *Pleurotheciella guizhouensis* is introduced herein as a new species.

#### Pleurothecium guangxiense

Taxon classificationFungiPleurothecialesPleurotheciaceae

G.L. Zhao, J.Y. Zhang & Y. Yang sp. nov.

ABB78751-984B-5DC4-9B70-F56629C4EB5F

Index Fungorum: IF905504

Fig. 3

##### Etymology.

Refers to the type locality, Guangxi Zhuang Autonomous Region.

**Figure 3.**
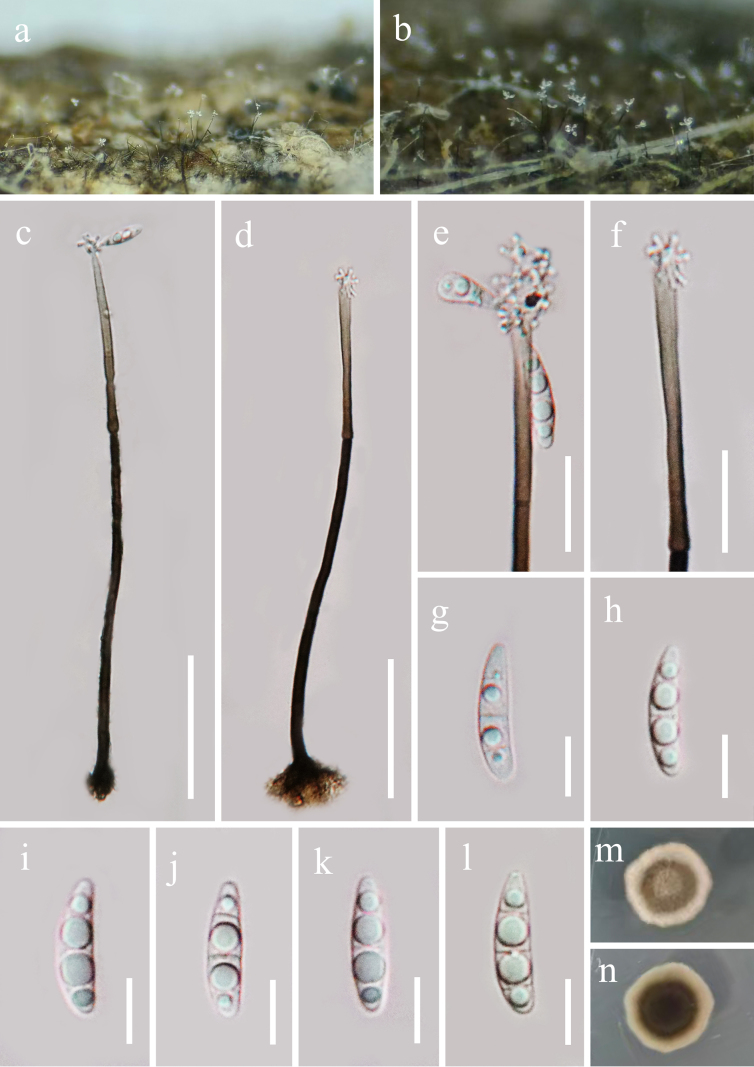
*Pleurothecium guangxiense* (HKAS 154366, holotype) **a, b**. Colonies on the host surface; **c, d**. Conidiophores, conidiogenous cells and conidia; **e, f**. Conidiogenous cells; **g–l**. Conidia; **m, n**. Surface and reverse colonies on PDA. Scale bars: 50 μm (**c, d**); 20 μm (**e, f**); 10 μm (**g–l**).

##### Holotype.

HKAS 154366.

##### Description.

***Saprobic*** on decaying wood in a terrestrial habitat. ***Sexual morph*** Undetermined. ***Asexual morph*** Hyphomycetous. ***Colonies*** on natural substrate superficial, effuse, solitary, brown to black, with white conidia aggregated at apex. ***Mycelium*** partly immersed, partly superficial, branched, septate, smooth-walled, brown to black hyphae. ***Conidiophores*** 174–245 μm long, 4.0–5.0 μm wide (*x̄* = 209 × 4.5 μm, n = 20), macronematous, mononematous, solitary, cylindrical, erect or slightly curved, unbranched, septate, dark brown at the base, becoming paler toward the apex, smooth. ***Conidiogenous cells*** 41–68 × 3.0–3.5 µm (*x̄* = 56 × 3.3 µm, n = 15), polyblastic, polyphialidic, integrated, terminal, smooth-walled, cylindrical, brown to hyaline, sympodial, with several cylindrical denticulate at apex. ***Conidia*** 18–24 µm long × 4.0–6.0 µm wide (*x̄* = 21 × 5.5 µm, n = 30), acrogenous, solitary, hyaline, straight or curved, guttulate, fusoid to clavate, 0–3-septate, rounded apical and tapering towards the base, wide in the middle, smooth-walled.

##### Culture characteristics.

Conidium germinated on PDA at 28 °C within 24 h from single-spore isolation. After two months of incubation at room temperature, the colony reached a diameter of 5 mm, dry, circular, irregular, smooth-edged, dense in texture, with a brown central region and an off-white outer margin, and the reverse was a slightly darker brown.

##### Material examined.

China • Guangxi Zhuang Autonomous Region, Qinzhou City, Pubei County (22°9'22"N, 109°22'41"E, altitude 63 m), on decaying wood in a terrestrial habitat, 5 October 2025, Gui-Li Zhao, G83A (HKAS 154366, holotype), ex-type culture GZCC 26-0126; *Ibid*., G83B (HKAS 154367, isotype), ex-isotype culture GZCC 26-0127.

##### Notes.

Phylogenetically, the two newly obtained strains GZCC 26-0126 and GZCC 26-0127 formed a distinct monophyletic lineage sister to *Pleurothecium pulneyense* (MFLUCC 16-1293), with 100% ML/1.00 BYPP support (Fig. 1). Pairwise nucleotide comparisons showed that *P. guangxiense* (GZCC 26-0126, ex-type) shared 802/823 bp (97.4%) sequence similarity in the LSU region, 453/454 bp (99.8%) in the SSU region, and 774/814 bp (95.1%) in the *rpb*2 region with *P. pulneyense* (MFLUCC 16-1293), excluding gaps. However, ITS sequence data for MFLUCC 16-1293 were unavailable. Morphologically, *P. guangxiense* differs from *P. pulneyense* in having shorter conidiophores (174–245 × 4.0–5.0 μm vs. 270–380 × 3.4–4.7 μm) and smaller conidia (18–24 × 4.0–6.0 μm vs. 23–30 × 7.0–8.4 μm) (Subramanian and Bhat 1989). Therefore, *Pleurothecium guangxiense* is introduced herein as a new species.

## Discussion

Guangxi and Guizhou, located in southern China, share a subtropical monsoon climate characterized by warm and humid conditions, complex karst landscapes, and diverse vegetation types (Hou et al. 2010; Kuo et al. 2011; Wang et al. 2022; He et al. 2025), which provide suitable ecological niches supporting high fungal diversity and abundant fungal resources (Wang et al. 2025b). Recent studies have provided increasing evidence that these regions represent hotspots for the discovery of novel fungal taxa, with numerous species recently reported from Guangxi and Guizhou provinces, China (Lu et al. 2018; Dong et al. 2023; Tang et al. 2023, 2024; Yang et al. 2023; Bao et al. 2025; Zhang et al. 2025a, 2025b; Zheng et al. 2025). In this study, two new species, *Pleurothecium guangxiense* from terrestrial habitats in Guangxi and *Pleurotheciella guizhouensis* from a freshwater habitat in Guizhou, are introduced based on morphological characteristics and phylogenetic evidence. The discovery of these two species further expands the known diversity of anamorphic taxa within *Pleurotheciaceae* and enhances our understanding of fungal diversity, ecology, and biogeography in southern China.

Before molecular data became widely available, the classification of hyphomycetous fungi was based on morphology, and it was not easy to classify taxa above the genus level unless they produced a sexual morph. However, it is now possible to place species within genera and higher taxa using DNA sequence data (Shenoy et al. 2007). Although species of *Pleurotheciella* share relatively similar sexual morphs and dactylaria-like polyblastic conidiogenesis with denticulate conidiogenous loci, substantial variation in their conidiophores and conidia makes these anamorphic characters particularly informative for species delimitation. Conidiophores exhibit a wide range of morphologies in natural habitats. Simple macronematous conidiophores are pigmented in *Pla. uniseptata* and *Pla. verrucosa* (Réblová et al. 2016; He et al. 2024), but hyaline in *Pla. ganzhouensis* and *Pla. irregularis* (He et al. 2024). In addition, fasciculate macronematous conidiophores are observed in species such as *Pla. guttulata* and *Pla. curvata* (Luo et al. 2018; Wang et al. 2025a), whereas synnematous conidiophores occur in *Pla. sympodia* (Wang et al. 2025a). Furthermore, conidiophores reduced to conidiogenous cells are present in several taxa, including *Pla. atroseptata*, *Pla. erumpens*, *Pla. xizangensis*, and *Pla. jiangxiensis* (He et al. 2024; Wang et al. 2025a). Conidia are likewise highly variable in shape, ranging from fusiform, cylindrical, and subclavate to capsule-shaped, meniscus-shaped, obovoidal, and distinctly U-shaped in *Pla. curvata* (Wang et al. 2025a).

The combined LSU, ITS, SSU, and *rpb2* phylogeny resolved *Pleurotheciella guizhouensis* and *Pleurothecium guangxiense* as two independent, well-supported lineages within their respective genera. Although these species share several morphological characteristics with related taxa, stable differences in conidiophore and conidial morphology, together with multilocus phylogenetic evidence, clearly distinguish them from their closest relatives. These findings are consistent with previous studies highlighting the importance of integrating morphological and multilocus phylogenetic evidence for species delimitation within *Pleurotheciaceae* (Réblová et al. 2016; He et al. 2024; Wang et al. 2025a).

Morphological variation within *Pleurotheciella* is relatively broad, whereas the generic concepts of *Pleurotheciella* and *Pleurothecium* overlap considerably in several diagnostic characters, rendering morphology alone insufficient for reliable species delimitation. Our results further demonstrate that an integrative taxonomic approach combining detailed morphological observations with multilocus phylogenetic analyses provides a robust framework for delimiting species and clarifying generic boundaries within *Pleurotheciaceae*.

## Supplementary Material

XML Treatment for Pleurotheciella guizhouensis

XML Treatment for Pleurothecium guangxiense

## Additional information

### Artificial Intelligence (AI) use

The authors accept full responsibility for the content of the manuscript, including the disclosure of any use of AI.

No AI tools were used in the preparation of this manuscript.

### Author ORCIDs

Y. Yang https://orcid.org/0000-0001-8268-487X

X.-J. Xiao https://orcid.org/0009-0003-8769-4534

Y.-Z. Lu https://orcid.org/0000-0002-1033-5782

K.D. Hyde https://orcid.org/0000-0002-2191-0762

Y.-P. Xiao https://orcid.org/0000-0003-1730-3545

G.-L. Zhao https://orcid.org/0000-0001-8370-4844

### Data availability

All of the data that support the findings of this study are available in the main text.
